# A Comprehensive Analysis of Gene Expression Changes Provoked by Bacterial and Fungal Infection in *C. elegans*

**DOI:** 10.1371/journal.pone.0019055

**Published:** 2011-05-13

**Authors:** Ilka Engelmann, Aurélien Griffon, Laurent Tichit, Frédéric Montañana-Sanchis, Guilin Wang, Valerie Reinke, Robert H. Waterston, LaDeana W. Hillier, Jonathan J. Ewbank

**Affiliations:** 1 Centre d'Immunologie de Marseille-Luminy, Université de la Méditerranée, Marseille, France; 2 INSERM, U631, Marseille, France; 3 CNRS, UMR6102, Marseille, France; 4 Institut de Mathématiques de Luminy, Marseille, France; 5 Department of Genetics, Yale University School of Medicine, New Haven, Connecticut, United States of America; 6 Department of Genome Sciences, University of Washington School of Medicine, Seattle, Washington, United States of America; Centre for Genomic Regulation, Spain

## Abstract

While *Caenorhabditis elegans* specifically responds to infection by the up-regulation of certain genes, distinct pathogens trigger the expression of a common set of genes. We applied new methods to conduct a comprehensive and comparative study of the transcriptional response of *C. elegans* to bacterial and fungal infection. Using tiling arrays and/or RNA-sequencing, we have characterized the genome-wide transcriptional changes that underlie the host's response to infection by three bacterial (*Serratia marcescens*, *Enterococcus faecalis* and *otorhabdus luminescens*) and two fungal pathogens (*Drechmeria coniospora* and *Harposporium* sp.). We developed a flexible tool, the WormBase Converter (available at http://wormbasemanager.sourceforge.net/), to allow cross-study comparisons. The new data sets provided more extensive lists of differentially regulated genes than previous studies. Annotation analysis confirmed that genes commonly up-regulated by bacterial infections are related to stress responses. We found substantial overlaps between the genes regulated upon intestinal infection by the bacterial pathogens and *Harposporium*, and between those regulated by *Harposporium* and *D. coniospora*, which infects the epidermis. Among the fungus-regulated genes, there was a significant bias towards genes that are evolving rapidly and potentially encode small proteins. The results obtained using new methods reveal that the response to infection in *C. elegans* is determined by the nature of the pathogen, the site of infection and the physiological imbalance provoked by infection. They form the basis for future functional dissection of innate immune signaling. Finally, we also propose alternative methods to identify differentially regulated genes that take into account the greater variability in lowly expressed genes.

## Introduction

*C. elegans* is now widely used as a model organism to dissect host innate immune responses to bacterial, fungal and viral pathogens [1], [2], [3], [4], [5], [6]. Studies on the transcriptional changes in *C. elegans* that accompany infection by different pathogens, often using microarrays, have identified many genes involved in the response to infection [7], [8], [9], [10], [11], [12], [13]. As the details are elucidated, the regulatory mechanisms that control these gene expression changes are turning out to be relatively complex. Several pathways have been shown to be involved, including the insulin-signaling pathway, the TGF-beta pathway and different MAPK cascades, with individual genes regulated by one or more pathways [3], [10], [12], [14], [15], [16], [17]. The precise mode of regulation depends on the tissue. In the major p38 MAPK pathway, for example, the p38 gene *pmk-1*, the MAP2K gene *sek-1* and the MAP3K gene *nsy-1* are crucial in both the intestine and the epidermis to counter infection. Only in the intestine, however, does activation of the pathway involve the protein kinase D gene *dkf-2* [18], [19]. Beyond this, several of the innate immune pathways have additional roles in development, or other aspects of organismal physiology. It appears that for some pathways, only certain elements are shared between the different functions. Thus, while a common TGF-beta ligand DBL-1 regulates both body size and the expression of *cnc* antimicrobial peptide genes, it does so through divergent pathways [20]. To give a second example, unlike *sek-1* and *nsy-1*, *pmk-1* does not have a role in neuronal development [21].

The complete understanding of this complexity requires comprehensively cataloguing the gene expression changes that accompany infection. In this study we have extended our previous analyses of the innate immune response of *C. elegans* and used tiling arrays and/or RNA-seq to characterize the transcriptional response of *C. elegans* to three bacterial and two fungal pathogens. These techniques have allowed us to define in far greater detail transcriptional responses to infection. We developed tools to allow the comparison of this new data to results from previous studies. These will be available to the research community, and will have a general utility for any study involving large numbers of genes. Using this tool, we were able to generate high-confidence lists of biomarkers. We have also defined groups of genes and functional annotation categories that are shared in the transcriptional response to more than one pathogen and groups of genes that are specifically regulated in response to only one pathogen, thereby directly contributing to the fuller characterization of innate immune responses in *C. elegans*.

## Results

### Comprehensive transcriptome data from worms infected with different bacterial and fungal pathogens

We previously characterized the transcriptional changes in host genes that accompany infection of *C. elegans* by the fungus *D. coniospora* or a number of different bacterial pathogens, using cDNA- and/or oligo-arrays [7], [11], [12], [22]. With the aim of obtaining a more comprehensive catalogue of the gene expression changes triggered by infection, as summarized in Table 1, we have now used tiling arrays and RNA-seq to extend those studies (Tables S1, S2, S3, S4, S5, S6, S7, S8). For samples assayed by these two methods, overall transcript expression levels were well correlated (Spearman r for uninfected nematodes and nematodes after infection with *S. marcescens, E. faecalis* or *P. luminescens* were 0.90, 0.88, 0.91 and 0.92, respectively) consistent with similar comparisons [23]. RNA-seq gives an unprecedented quantitative measure of transcript levels, and very low technical variability [24], [25]. In situations where there is little biological variation, a single sample may suffice to obtain robust genome-wide information. In the present study, despite the known experimental variation in the response to infection ([22]; see below), while tiling arrays were done on two or three replicates (see Methods), practical constraints limited our RNA-seq analyses to single biological samples. We therefore focused our initial analysis of the RNA-seq data on the identification of genes identified as differentially-regulated in more than one infection model.

**Table 1:** Techniques and sources for transcriptome studies.

	cDNA-array	Oligo-array	Tiling array	RNA-seq
*Serratia marcescens*	Mallo et al.[11]	Wong et al.[22]	This study, Table S1	This study, Table S2
*Enterococcus faecalis*		Wong et al. [22]	This study, Table S3	This study, Table S4
*Photorhabdus luminescens*		Wong et al.[22]	This study, Table S5	This study, Table S6
*Drechmeria coniospora*	Pujol et al.[12]	Pujol et al. (unpublished); Table S17		This study, Table S7
*Harposporium* sp.				This study, Table S8

### Comparison of the genes transcriptionally regulated upon bacterial infection by RNA-seq

**Figure 1:**
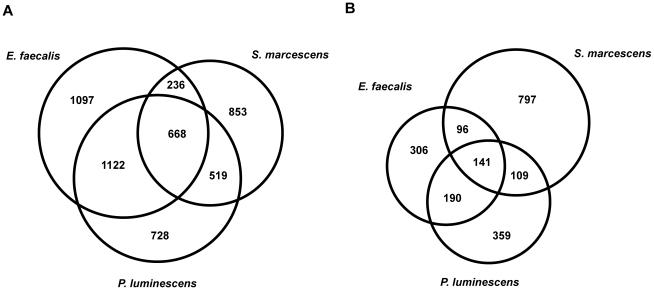
Genes regulated by bacterial infection. Proportional Venn diagrams showing the overlap of genes up-(A) or down-regulated (B) by *S. marcescens*, *E. faecalis*, *P. luminescens* using RNA-seq.

The bacterial pathogens *S. marcescens*, *E. faecalis* and *P. luminescens* all colonize the intestine of *C. elegans*. Infection with each of the bacteria caused the expression of between 2,000 and 3,000 genes to be up-regulated (Table S9A), using simple percentile-based criteria (see Methods). The largest overlap was between the genes induced by *E. faecalis* and *P. luminescens*, which included 1,122 genes that were not up-regulated by *S. marcescens* (Figure 1A, Table S10A). Among the 668 genes up-regulated by all 3 bacterial pathogens (Table S10B), there were multiple C-type lectins, which are a common component of the response of *C. elegans* to many pathogens [26]. We also found several genes known to be involved, or putatively involved, in glycoprotein biosynthesis, including *bah-1*, *bus-1*, -*8*, -*18*, and -*19*, as well as *bre-1*. Mutation in any of these genes leads to resistance to certain bacterial infections, via changing surface epitopes targeted by pathogens. For example, *bah-1* is required for *Yersinia* biofilm attachment to the cuticle [27]. There were also 13 F-box containing proteins; these have been suggested to be ubiquitin ligases involved in host defense [28], as well as 11 cytochrome P450 genes which are expected to function in the detoxification of xeniobiotics [29]. In addition, 9 hedgehog-like proteins, possibly involved in signaling, were identified, as well as the ERK family MAPK gene *mpk-2.* This latter gene is also up-regulated by *M. nematophilum*, *P. aeruginosa* and *S. aureus* infection [13], [30]. Interestingly, although the structurally-related kinase MPK-1 has been shown to be important for the rectal epithelial cell swelling response to *M. nematophilum* [31] and for the swelling response to *S. aureus*, it is dispensable for the intestinal transcriptional response [30]. Our results identify *mpk-2* as a candidate regulatory component of the immune response in the intestine.

The comparisons of individual up-regulated genes are relatively stringent. To complement these analyses, we also used overrepresentation of gene categories within the lists of differentially regulated genes, using EASE [32]. Using class testing with our up-to-date bibliographic annotation list (see Methods), we found genes regulated by diverse stresses (cadmium, high salt, and the polychlorinated biphenyl PCB52) to be enriched in genes up-regulated by all three bacterial pathogens. Recently, the transcription factor *zip-2* was shown to be involved in the regulation of defense genes in the intestine of *C. elegans* [33]. Interestingly, targets of *zip-2* were also enriched in the genes up-regulated by all three bacterial pathogens, suggesting that it is important for the transcriptional response to various intestinal pathogens. On the other hand, only in the *S. marcescens* and *P. luminescens* induced gene sets did we find an overrepresentation of targets of the DKF-2-, p38 MAPK- and TGF-beta pathways (Table S11A).

With regards to genes down-regulated after infection, again by percentile-based criteria, there were around one thousand after each of the three bacterial infections (Figure 1B, Table S10C). Some 140 genes were down-regulated by all three bacteria, including *fip-5* and four genes belonging to the *nlp*-family (reflected by the overrepresentation of the class “defense response to fungus” [GO:0050832]). Otherwise, there was a common decrease in the expression of ribosomal genes and genes involved in protein translation and transcription (Table S10D), indicative of the marked changes in cell physiology that accompany infection. Class testing of the genes down-regulated by the three bacteria using our bibliographic annotation list revealed an enrichment in genes down-regulated by *M. luteus, Pseudomonas sp.* and *B. megaterium* and genes regulated by *daf-2* and *daf-16*, by osmotic stress and by cadmium (Table S11B), presumably reflecting the fact that infection provokes cellular damage.

### Comparison of the genes transcriptionally regulated upon fungal infection by RNA-seq

In addition to the bacterial data sets, we also collected RNA-seq data from samples of worms infected with two fungal species, *D. coniospora* and *Harposporium* sp. Unlike *D. coniospora* that infects *C. elegans* via its cuticle and epidermis [34], the hook-like spores of this second fungus are able to enter into the intestine of nematodes, germinate and invade the host's tissues, before emerging through the epidermis [35]. They then form new spores, thus completing the infectious cycle. The strain of *Harposporium* we used (JUf27) was isolated from an infected *C. elegans* individual found in a rotting apple collected in Orsay (Essonne), France, in October 2008. It is therefore likely to be a natural pathogen of *C. elegans*.

**Figure 2:**
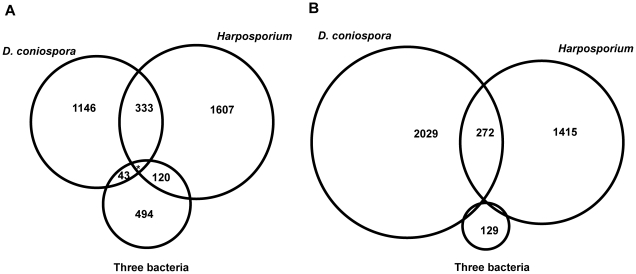
Genes regulated by fungal or bacterial infection. Proportional Venn diagrams showing the overlap of genes up-(A) or down-regulated (B) by *Harposporium* sp., *D. coniospora* and three bacteria (common to *S. marcescens*, *E. faecalis* and *P. luminescens*) using RNA-seq. The asterisk indicates that 11 genes were up-regulated by *Harposporium* sp., *D. coniospora* and three bacteria; 6 genes were down-regulated by the three bacteria and either *Harposporium* sp. or *D. coniospora*.

When we compared gene expression profiles of *C. elegans* infected with *D. coniospora* and *Harposporium*, we found that close to 1,500 and 2,000 genes were up-regulated, respectively, and similar numbers of genes were down-regulated (Figure 2, Table S12A, B). Of the 333 genes induced by both fungi (but not the three bacteria) (Figure 2A), there were many genes encoding proven or potential antimicrobial peptides, including *abf-1* [36], multiple *cnc*, *fip*, *fipr* and *nlp* genes [12] as well as 8 nematode specific peptides [28] of the a, c, d and e classes. Additionally, there were four genes encoding insulins, including the well-characterized DAF-2/DAF-16 pathway agonist *ins-7* (Table S12D) [37], [38]. There was also an overrepresentation among the genes commonly up-regulated by the two fungal pathogens of the categories “genes down-regulated in *daf-16”* and “up-regulated in *daf-2*” (Table S13A). Taken together this highlights the importance of the DAF-2/DAF-16 pathway and insulin signaling in *C. elegans* anti-fungal defenses. The DAF-2/DAF-16 pathway is also involved in resistance to multiple environmental insults. We found an overrepresentation in the shared fungal response genes of genes reported to be up-regulated by diverse stresses, including high salt, heavy metals and the bacterial pore-forming toxin Cry5B ( Table S13A). The overlap, however, between the individual genes in each of these categories and those identified as targets of insulin signaling was limited (a maximum of 13%), emphasizing that other pathways are also involved in the organismal response to biotic and abiotic stress.

One relatively frequent theme among the 272 genes that were down-regulated by the two fungal pathogens (Figure 2B, Table S12B) was a link to feeding behavior. For example, dopamine signaling affects locomotory slowing in response to food [39], and we found both *cat-2* (dopamine biosynthesis) and *dop-1* (dopamine receptor). We also found *dgk-1*, whose activity regulates locomotion, too. In addition, we identified 3 of the nematode's globin-like genes, some of which are known to be expressed in the sensory neurons and linked to oxygen sensing, which is an integral part of the detection of food, as well as *eat-18*, a member of the gustatory class of receptors, and *daf-28*, which can be considered as a starvation response gene [40] (Table S12F). This supports the existence of a possible link between metabolism and innate immune regulation [41].

### Comparison of the genes transcriptionally regulated upon bacterial or fungal infection

Overall, and confirming the trend noted above, there was an inverse relationship between the categories of genes regulated upon infection by both fungi as opposed to those regulated following bacterial infection, such that there was an overrepresentation of genes repressed after infection by *S. marcescens*, *E. faecalis* and *P. luminescens* among the genes induced by both *D. coniospora* and *Harposporium*, and of genes up-regulated upon infection by each of the bacterial pathogens among the genes down-regulated upon infection with both fungal pathogens (Table S13).

We also established the list of genes regulated by all five pathogens, both fungal and bacterial. There were no commonly down-regulated genes, and only 11 up-regulated genes (Figure 2; Table S12A, B). Remarkably, absolutely nothing is known about 7 of them, illustrating how incomplete our understanding of the innate immune response is. For the remaining 4, they are characterized only by their structure: 1 cytochrome P450, 1 zinc finger protein, 1 tetraspanin and 1 F-box protein (Table S12C).

When we looked for overlaps in annotations in the categories of up-regulated genes, we found themes related to organismal stress responses, such as induction by exposure to cadmium and a compromised cuticle, in addition to an overrepresentation of genes regulated by *pmk-1* or by infection by *M. nematophilum, P. aeruginosa* and/or *S. aureus* (Table S13A). This reinforces a trend previously reported [22].

Both the three bacteria we used and *Harposporium* infect *C. elegans* via the intestine. The number of genes induced by these 4 pathogens (120 genes) is much greater than the number of genes induced both by the three bacteria and the epidermal pathogen *D. coniospora* (43) (Figure 2A, Table S12A). There was gene expression pattern data for 21 of the 120 genes in WS210, and 9/21 were reported to be expressed in the intestine (43%). This is markedly higher than for the group of 43 genes induced by *D. coniospora* and the three bacteria, where only one of the 9 genes for which there was data was reported to be expressed in the intestine (11%). Additionally, in both the *Harposporium*- and the bacterially-induced genes there was an enrichment of genes identified as intestinally expressed, based on mRNA tagging [42]. This prevalence of intestinally expressed genes presumably reflects the fact that *Harposporium* and the three bacteria establish an intestinal infection, as opposed to *D. coniospora*, which infects via the epidermis. The genes induced by intestinal pathogen-infection included genes with known involvement in the response to intestinal infection, e.g. eight C-type lectins and the lysozyme *lys-2*. In addition, we found the trypsin-like proteases *try-3* and *try-7*, which have not yet attracted attention as putative defense effectors, as well as the ERK MAPK gene *mpk-2*, which as noted above is a candidate regulator of the intestinal response (Table S12E). Gene categories shared between the genes up-regulated by the intestinal pathogens also included some known to be involved in the intestinal immune response, e.g. targets of the p38 MAPK pathway, regulated by the transcription factor *zip-*2 or by *dbl-1* (Table S13A). Furthermore, we found an enrichment in genes down-regulated in *daf-2* mutants, again underscoring the general importance of insulin signaling in the interaction between *C. elegans* and pathogens.

There were just six genes down-regulated by all 4 intestinal pathogens; the only one functionally annotated was the histone H2B *his-8* (Table S12G). Interestingly, when we considered categories, the genes down-regulated by the intestinal pathogens were enriched for genes up-regulated after infection with the epidermal pathogen *D. coniospora* (Table S13B). There was equally an enrichment for genes up-regulated after infection with either of the fungal pathogens among the genes commonly down-regulated by all the bacterial pathogens (Table S11B). This supports the notion that the repertoires of genes involved in the response to each pathogen depends both on the nature of the microbe and the site of the infection.

### Comparison of peptide length

**Figure 3:**
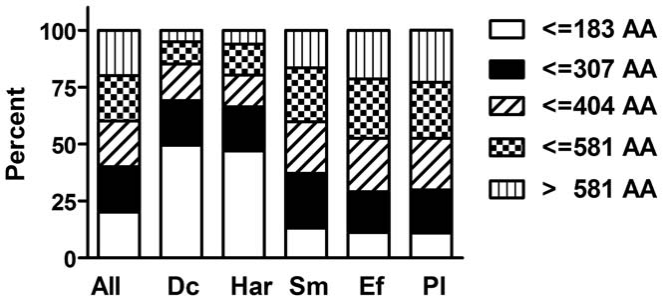
Peptide length of induced transcripts. Transcripts up-regulated upon fungal infection are on average shorter than those induced by bacterial infection. All transcripts were divided into five classes based on the length of the encoded proteins as shown (AA: amino acid). Bar diagram showing the percentages of transcripts coding for proteins with a certain length in the list of all transcripts and in the lists of up-regulated transcripts for different pathogens (RNA-seq). All: all transcripts; Dc: *D. coniospora*; Har: *Harposporium* sp.; Sm: *S. marcescens*; Ef: *E. faecalis*; Pl: *P. luminescens*.

During our analyses, we realized that there were differences in the median size of the peptides and proteins up-regulated upon fungal or bacterial pathogen exposure. Fungal infection was associated with a greater proportion of smaller peptides and proteins; the percentage of peptides with less than 90 residues was significantly higher in these samples compared to those from bacterially infected worms (p<0.0001, Chi-square test). This difference in relative frequencies was still apparent when we defined five equal classes of peptides based on their length (Figure 3). Clearly, infection with *D. coniospora* or *Harposporium* induced more genes that code for smaller proteins. Prominent among those induced by both fungi, as noted above, were several classes of antimicrobial peptide genes, as well as the 4 insulin-like genes, *ins-7*, *ins-11*, *ins-23* and *ins-36*. These results reinforce the notion that part of the response to infection in *C. elegans* may be dependent upon the nature of the microbe, regardless of the site of the infection.

### Conservation of infection-regulated genes

When comparisons are made across a broad range of host species, effectors of innate immunity are often found to be highly divergent, probably as a result of strong selective pressure. For example, many gene classes important in *C. elegans* are absent from the plant pathogenic nematode *Meloidogyne incognita* [43]. Even within the genus *Caenorhabditis*, detailed locus-specific studies have shown that there are marked differences for some categories of defense genes, including the inducible *nlp* and *cnc* genes, which have undergone multiple duplications in *C. elegans* compared to *C. remanei* or *C. brenneri* [12]. To look in a systematic way at defense gene conservation, we turned to the cisRED database [44]. This lists transcripts for which there are orthologues in at least 3 of the species *C. briggsae*, *C. remanei*, *C. brenneri*, *C. japonica*, *Pristionchus pacificus*, *Brugia malayi*, and *Trichinella spiralis*. These transcripts correspond to 3,421 *C. elegans* genes, or 18.4% of the total set of genes detected by RNA-seq in uninfected worms. When we calculated the percentage of genes conserved among the genes up-regulated after infection with any of the five pathogens, we found that it was significantly lower (10.9% for *D. coniospora,* 12.6% for *Harposporium,* 13.4% for *E. faecalis,* 14.7% for *S. marcescens,* 15.5% for *P. luminescens,* p<0.0001 for all comparisons, Chi square test). Although these figures cannot be entirely trusted, since the current genome sequences of the other nematode species are not complete, this is further evidence for the unusually rapid evolution of defense genes.

### WormBase Converter: A tool for accurate comparison of lists of *C. elegans* genes

**Figure 4:**
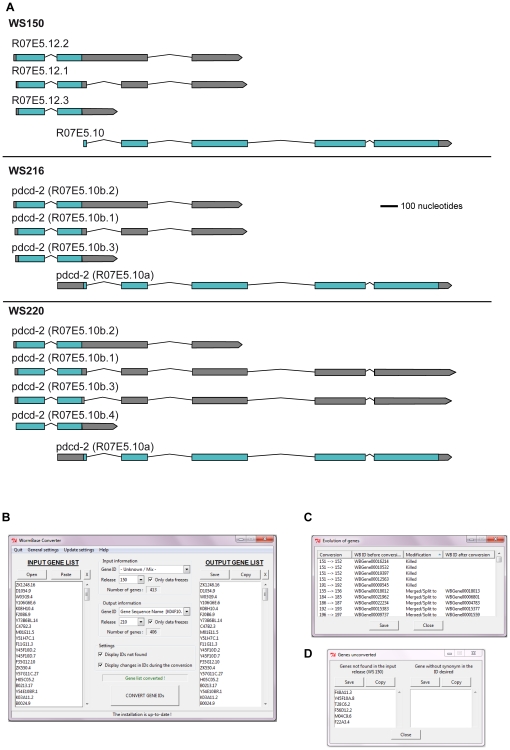
The WormBase Converter. Constant changes in gene annotations create the need for WormBase Converter. (A) Example of the gene R07E5.12 that has undergone a merge with R07E5.10, which then has been re-annotated. More recently, as seen here in WS220, the structure of R07E5.10 has been further modified. (B) Example of an input list with CDS Sequence names as identifiers based on WormBase version WS150 and the corresponding output list in WS210 using gene sequence name as identifier. The output list can be copied and pasted into any spreadsheet for further use. The number of successfully converted genes is indicated. In this case, 11 out of 413 genes were absent from the output list; 5 had been “killed”, and 6 not found in the WS150 release. On the other hand, because of gene splits, the output list contained 4 new genes. (C) List showing the changes that occurred during the conversions, the nature of the change and the version when the change was implemented, including the 5 “killed” genes. (D) List of 6 genes that were not found in the version used as input.

We then wished to compare our new RNA-seq data with the results from the tiling arrays and with our previous transcriptome studies. As is a general rule for *C. elegans*, the interpretation of our previous studies was based on gene models and functional annotations present in the nematode-specific database WormBase at the time [45]. Gene annotations are subject to extensive changes as the knowledge of gene structure and function progresses. For example, between the WS150 release (November 2005) and WS210 (March 2009) the structure of more than 650 genes has been altered, including 20% of cases where a gene was completely removed. As an illustration, in the WS152 release, the gene R07E5.12 was merged with R07E5.10, and the composite gene retained the name R07E5.10 (Figure 4A). Published experimental results that predate a change in annotation can therefore refer to genes that no longer exist. As an illustration (Figure S1), the microarray probe cea2.i.18533 was associated with the gene C03B8.1 until WS216. The gene C03B8.1 was then merged into the gene C03B8.3. As a consequence, the microarray probe cea2.i.18533 will be associated to the gene C03B8.3 from WS217 on. Although this change is recorded in WormBase (Figure S1), the obsolete gene name C03B8.1 does figure in older publications. This clearly renders any direct comparison of analyses from different studies problematic. Cross comparisons of published data sets are frequently further hampered by the use of different gene identifiers (e.g. gene sequence name versus transcript sequence name), and the absence of a clear indication of the WormBase version used. Therefore, we first developed a tool, WormBase Converter (publicly available at http://wormbasemanager.sourceforge.net/), that enables the conversion of lists of genes with different identifiers and from different WormBase versions to a single coherent format, using the WB ID, referenced to a defined version of WormBase. The WB IDs are linked to the physical coordinates in the genomes, as are microarray probes and RNAi clones, so that these automatically follow evolutions in gene structure. If a gene has changed, the output list given by the Wormbase Converter will contain the new name (Figure 4B). The tool further indicates all changes that occurred between the different versions of WormBase and thus makes it possible to track down genes that have been deleted from the current version of WormBase, that have been merged with other genes and so on (Figure 4C). It also gives the names of genes that were not found in the selected version of WormBase (Figure 4D). To avoid the necessity of individual laboratories to maintain up-to-date and manually-corrected conversion files, we also provide access to Wormbase Converter at http://www.ciml.univ-mrs.fr/applications/WB_converter/.

### Cross-platform comparison of transcriptionally regulated genes

We first used WormBase Converter to generate coherent data sets for our results from RNA-seq and cDNA-, oligo- and tiling arrays, with the WormBase WS210 release as our reference. We applied the same broad definition, i.e. the top or bottom 18.75th percentile of differentially-expressed genes (see Methods for details), to identify infection-regulated genes (Table S9). Our most complete set of data concerns transcripts from worms infected with the Gram-negative intestinal pathogen *S. marcescens*. As mentioned above, transcript expression levels were well correlated between tiling array and RNA-seq data sets. When we compared genes identified as up-regulated by both techniques the overlap was 44% (Table S14A). When we compared the new RNA-seq and tiling array data with our previous cDNA- and oligo-array data (Tables S14, S15A), there was a comparable concordance between those genes identified as up-regulated by cDNA- and tiling arrays (33%) or RNA-seq (34%). This is encouraging as not only were different techniques applied, but the biological samples were completely distinct, having been prepared at a number of years' interval by different investigators. As discussed further below, the results from the oligo-arrays stood out for their relatively poor degree of overlap with the results from any of the three other techniques (from 4% to 14%) and were excluded from further analyses. Of the 55 genes found to be up-regulated by both cDNA- and tiling arrays, 44 (80%) figured in the list of up-regulated genes based on RNA-seq. As expected, among the genes up-regulated in cDNA-, tiling arrays and RNA-seq, there were many encoding collagens, as well as lectins and lysozymes (Table S15B). Establishing the subset of genes identified as differentially regulated by several different techniques can compensate for the limitations inherent to each technique, and the biological variation in different experiments. The list of genes identified as up-regulated by several techniques therefore represent a high-confidence catalogue of infection-regulated genes that could be used as biomarkers for *S. marcescens* infection (Table S15B). Because cDNA-arrays are biased to highly expressed genes, the genes in this intersection are all relatively highly expressed (Figure S2A). When the overlap is not constrained by the cDNA-array data, this bias disappears (Figure S2B). Because lowly expressed genes are of potential interest, we generated a second list of biomarkers that are expressed at low levels in the absence of infection, but show a strong degree of induction (arbitrarily defined as>20 fold induction) (Figure S2C, Table S15B). Comparison of data sets from samples infected with *E. faecalis* or *P. luminescens* generated using RNA-seq, oligo- and tiling arrays yielded similar results (Tables S14, S16), and could be useful for anyone wishing to generate lists of biomarkers relevant to these pathogens.

We then compared the genes up-regulated during the response of *C. elegans* to infection with the fungus *D. coniospora* in RNA-seq, cDNA-arrays and oligo-arrays (Table S17). Almost half of the genes (11/25) identified by 2 or more techniques belonged to the two classes of antimicrobial peptides (NLP and CNC) that we have shown most clearly to be involved in the antifungal immune response of *C. elegans*. They exclusively figured in the list of genes identified as up-regulated by all techniques. This validates the use of multiple techniques to identify high-confidence candidate genes. Otherwise, as discussed below, the overlap of genes commonly up-regulated in the different data sets was comparatively small (Table S18).

## Discussion

In this study, we have undertaken an extensive characterization of the changes in gene transcription in *C. elegans* after infection with three bacterial and two fungal pathogens. We have confirmed genes and pathways shown to be involved in the immune response of the nematode in previous studies and extended the lists of genes that are up-regulated or down-regulated in response to infection with single or multiple pathogens. Furthermore, we have established lists of genes induced by bacterial versus fungal and intestinal versus epidermal infection. These lists characterize pathogen-class and tissue specificity of the immune response. This comparative transcriptomic study reveals the complex composition of the immune response of the nematode.

### Comparison of transcriptional changes induced by different pathogens

Large-scale transcriptome studies suffer from variability linked to the experimental technique chosen, and even to the person carrying them out. Our homogenous transcriptome data sets allowed us to perform meaningful inter-pathogen comparisons and define genes induced as part of a shared response. Importantly, the RNA-seq data also allowed us for the first time to compare the responses to two fungal pathogens. Through a comparative analysis with our previous microarray data, we could also identify with high confidence genes specifically induced by one pathogen. This is important as experience has shown that the transcriptional response to infection is quite variable from one experiment to another. There are several reasons for this. One is that many of the genes regulated upon infection are also influenced by other environmental factors. A clear example is the expression of the gene *nlp-29* which is partially dependent upon the osmolarity of the culture medium [12], [22], which therefore needs to be carefully controlled. Secondly, microbial pathogenicity is also highly dependent on the precise culture conditions; there is not always a simple way to control tightly all the relevant parameters, especially since some are not even known. Thirdly, there is a dynamic interaction between host and pathogen, with the host response influencing pathogen virulence and vice versa. Given the intrinsic variability on both sides, subtle differences at the start of an infection can lead to marked dissimilarities in its progression. In the current analysis, the results of our previous study of the response to *S. marcescens* using oligo-arrays [22] stood out. As this was not the case for the results for the response to 2 other bacterial pathogens, and given the underrepresentation in the *S. marcescens* data set of “common response genes” [22], this presumably reflects an experimental difference in the strength of the infection for the samples prepared for analysis using oligo-arrays.

A striking observation from the RNA-seq data was that among the genes commonly regulated upon infection by both *D. coniospora* and *Harposporium* there was a subset of genes that was regulated inversely after bacterial infection. Thus, we found around 80 genes up-regulated by both fungi but down-regulated by *E. faecalis*, *P. luminescens* or *S. marcescens*, and conversely there was an enrichment in bacterially-induced genes among the genes down-regulated upon infection with both fungal pathogens. In the former category, there were members of several antimicrobial peptide families, which explains in part the surprising bias towards shorter proteins in the shared fungal response. Another class that contributed to this bias was insulin-like molecules. The potential role of insulin signaling in regulating the response of *C. elegans* to fungal pathogens merits further study.

The neprilysin class of M13 peptidases is another example of a gene class differentially regulated between fungal and bacterial infection. Neprilysins are zinc metallopeptidases, generally found on the outer surface of animal cells. They cleave small signaling peptides (e.g. enkephalin, tachykinin and insulin) and thereby block their action. Of the 27 members in the *C. elegans* genome, 13 were down-regulated by *D. coniospora* or by *Harposporium*, whereas only 2 were induced. In contrast, one or more of the 3 bacteria led to an up-regulation of 9 of the M13 peptidases and to a down-regulation of 2. Only 2 members of the family in *C. elegans* have been well-characterized functionally, *nep-1* and *nep-2*. Neither were differentially regulated. On the other hand, 6 of the M13 peptidase genes that exhibited differential expression were part of a cluster of 8 peptidase genes on the left arm of chromosome II. They may form part of a regulatory circuit governing autocrine or paracrine small peptide signaling, an aspect of the immune response that is currently poorly understood.

It is, however, important to bear in mind that if one tissue is the main site of the organism's transcriptional response to infection, measured changes in gene expression in different tissues may reflect the normalization procedure. To put it another way, in the case of RNA-seq, given that the absolute number of reads for a particular transcript is divided by the total number of aligned reads obtained with that sample, if there is intense transcriptional activity in the intestine, then the transcript level for epidermal genes will be artificially depressed. This could contribute to the observed down-regulation of the epidermally-expressed *fip* and *nlp* genes after infection with any one of the three bacterial intestinal pathogens. Since some intestinal genes like *mtl-1* and *-2* are also down-regulated upon bacterial infection, this cannot explain all observed transcriptional changes. Indeed, there are now examples of pathogens triggering down-regulation of host genes (e.g. [48]); this is clearly an area that merits further investigation.

### WormBase Converter

The comparison of our new data sets to previous microarray studies proved surprisingly complicated, due to the use of different gene identifiers and WormBase versions. While the online tool WormMart [49] is useful for changing identifiers within a single WormBase release, it is not designed to convert lists between releases. Furthermore, we occasionally found irrelevant genes in the output from WormMart using WS210, because of intrinsic errors in the way queries were handled by WormMart (I. Engelmann, unpublished observations). In developing the WormBase Converter, we circumvented these problems.

While at first sight this might appear a simple task, the nature of gene annotation changes makes following the evolution of gene structures surprisingly complex. In order to permit accurate cross-release comparisons, WormBase Converter has to take into account all successive changes in gene annotation and modifies the list of gene identifiers accordingly. Using this tool we became aware of a very occasional problem with WormBase data. Although the terminology applied to annotation changes, made manually by WormBase curators, should be invariant, this is not absolutely always the case. Thus, for example, while “split from” should mean that a new daughter gene is formed in part from an original parent gene, with retention of a modified parent gene, there are rare examples where during annotation the modified parent gene is deleted, without formally having been “killed”. Obviously, these odd database inconsistencies cannot be taken into account automatically by a tool like the WormBase Converter, which needs to run with fixed rules, but such rare inconsistencies can be readily identified and we included the option to correct manually the output files from the WormBase Converter.

One other minor limitation to the WormBase Converter is that no conversion of a list of gene names into transcript names is possible. This is a consequence of the intrinsic organization of WormBase data, since changes to gene structure between different releases are recorded using the WB ID identifier, which is a gene level identifier [45]. On the positive side, our program has been written to maintain lists continuously up-to-date, now and in the future, which is important since new WormBase releases are scheduled every month. Since it is written as both a stand-alone and a server-based application, and can be run under Windows, Mac OS or Linux, we hope that it will be of widespread utility to laboratories dealing with lists of *C. elegans* genes, as well as helping to improve the standard of consistent gene annotation.

### Alternative methods to define up- or down-regulation

Most studies define up- or down-regulated genes on the basis of fold change in expression between two conditions. It remains a reasonable criterion for comprehensive comparisons of gene lists, and we therefore used it above to highlight commonalities and differences in the response of *C. elegans* to different pathogens. The extended dynamic range of RNA-seq, however, potentially biases results based on fold change, as there is a greater relative variation in expression for lowly expressed genes [46], [47]. The fold change-based approach can thus yield a high proportion of false positive candidates in the area of low expression where the variability of expression is high for technical reasons.

Since RNA-seq gives transcript numbers as a read-out, one could alternatively calculate the absolute change in transcript numbers between two conditions (transcript number in condition A minus transcript number in condition B). But from a biological perspective, it is not at all obvious that change in absolute transcript number is a meaningful measure when looking across large sets of genes with an enormous range of expression levels. For example, one can imagine a case where there are 1000 transcripts of gene X under condition A, and 1100 under condition B; for gene Y there are 10 and 110, respectively. In both cases the change in absolute number is the same (100), but for gene X, the relative fold change is 1.1 and for gene Y it is ten-fold higher. One might expect the change in Y to have more biological impact.

Alternative methods have been developed but these all use either the Poisson distribution or Fisher's exact test to model data, neither of which cope well with biological variation [50].

**Figure 5:**
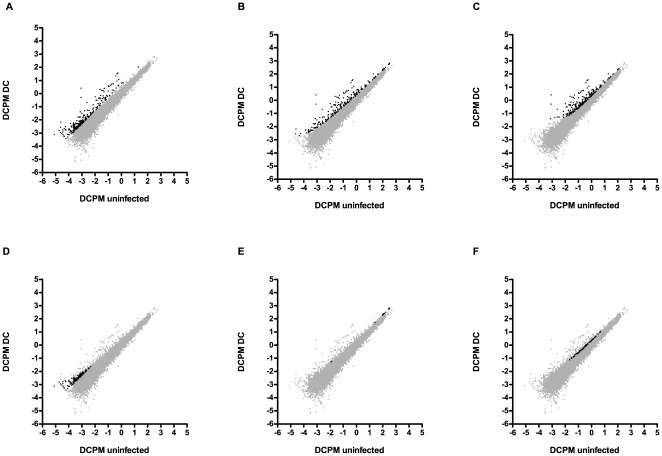
Selection of differentially-regulated transcripts. Dot plots showing log10 transformed dcpm values for expression of transcripts in uninfected (x-axis) versus *D. coniospora* infected worms (y-axis). Transcripts identified as up-regulated by one or more methods are shown in black, the others are shown in grey. (A) transcripts defined as up-regulated if the log2 fold change is greater than the 97th percentile of all up-regulated transcripts; (B) transcripts defined as up-regulated by alternative approach 1 (see Methods); (C) transcripts defined as up-regulated by alternative approach 2 (see Methods); (D) transcripts only defined as up-regulated if the log2 fold change is greater than the 97th percentile of all up-regulated transcripts and not by either of the other methods; (E) transcripts only defined as up-regulated by alternative approach 1; (F) transcripts only defined as up-regulated by alternative approach 2.

We therefore experimented with two alternative approaches (see Methods and Figure S3 for details). Both approaches identify differentially regulated genes on the basis of comparisons with genes of similar levels of expression under control conditions and additionally take into account variability at the lower end of the expression level in order to minimise false-positives. The two different approaches give overlapping but non-identical results (Figure 5, Figure S4). Inspection of the lists of genes that are identified using each method suggests that, depending on the purpose, one approach may be more appropriate. Thus, though the methods look promising, they clearly require further validation with data sets from a broad range of biological samples. Neither method, however, provides a solution for the correct quantitative analysis of transcripts with no detectable expression in one of the two conditions, as their ratio is either zero or infinity. These can, however, merit special attention. In our study, transcripts with no expression in the uninfected condition, but which are induced upon infection, could be considered the most infection-specific and the corresponding genes potentially interesting ones. We found 619 such genes (in WS210), including *abf-4*, *cnc-5, cnc-9*, *fipr-8*, *fipr-23*, *ins-8, ins-32 and ins-39* and several nematode-specific peptides. Remarkably, only 4 of the 619 genes figure in the list of genes identified by oligo-array as being induced by *D. coniospora* infection (data not shown). Methods based on differences in transcript counts would allow these genes to be identified, otherwise, another way to address this issue is to set the value for the condition with no expression to some arbitrary small value [24]. This allows such transcripts to be included in the normal analysis scheme, but their calculated fold change will be entirely artificial. Clearly, other approaches to analyze data with linear values and zero expression in one condition are needed.

### Conclusions

This study has greatly expanded our understanding of the molecular repertoire of the *C. elegans* genes that are involved in the transcriptional response to infection. Extending previous observations, we have shown that not only is the host response composed of both pathogen-specific and pathogen-shared elements, but that the latter depends upon the site of infection. To enable this work, we needed to construct bioinformatic tools, which we expect to be of broad utility to the research community, especially as with the advent of improved sequencing methods, the predicted structures of genes are constantly evolving.

## Methods

### Bacterial strains and culture

*P. luminescens* strain Hb was cultured in liquid LB medium at 30°C overnight, spread on NGM plates, incubated for 24 h at 30°C, then another 20 h at 25°C. *E. faecalis* strain OG1RF was cultured in liquid BHI at 37°C overnight, spread on NGM plates, incubated for 24 h at 37°C, then cultured another 20 h at 25°C. OP50 was cultured in liquid LB medium at 37°C overnight, spread on NGM plates, incubated for 24 h at 37°C, then cultured another 20 h at 25°C. *S. marcescens* strain Db10 [51] was cultured in liquid LB at 37°C overnight, put on NGM plates for 24 h at 25°C, then cultured another 20 h at 25°C.

### Infection

Eggs were allowed to hatch in the absence of food at 25°C (in the case of N2 used for bacterial infections) or 15°C (*fer-15* for fungal infections). Synchronized L1 larvae were transferred to NGM agar plates spread with *E. coli* strain OP50. N2 worms were cultivated at 25°C until the young adult stage (43–45 h) and then transferred to plates spread with *S. marcescens*, *E. faecalis* or *P. luminescens* and cultivated at 25°C for 24 h. For *D. coniospora* and *Harposporium* sp. infection, *fer-15* worms were cultivated at 25°C until the L4 stage (36 to 38 hours) before being exposed to fungal spores. Nematodes were harvested after 12 hours for *D. coniospora* and 24 hours for *Harposporium* sp. This time-point was chosen for *Harposporium* sp. as the infection proceeded at roughly half the rate as seen with *D. coniospora* (C. Couillault, personal communication). Worms were harvested and washed twice in M9. As far as possible, larvae were separated from adults during washing. The volume of the pellet was measured and 10 volumes of TRIZOL (Gibco BRL) added. Tubes were vortexed for 45 sec, placed in liquid Nitrogen, flash frozen completely, then thawed completely in a 37°C water-bath. This freeze/thaw process was repeated 4 times. The homogenate of worms and Trizol was frozen at −80°C and sent to the Reinke laboratory (Yale, New Haven) on dry ice.

### RNA preparation, microarray and RNA-seq analyses

cDNA was generated from extracted polyA RNA following standard techniques as described elsewhere [23]. One replicate was used for RNA-seq for each condition. Two biological replicates were used for tiling arrays of *E. faecalis* infected nematodes and three replicates were used for tiling arrays for worms infected with *S. marcescens* and *P. luminescens* as well as for control samples. Tiling arrays were processed and analyzed as previously described [23]. Briefly, data were analyzed by first computing the difference between perfect match and mismatch (PM – MM) values for each probe. Experiments were conducted in triplicate or duplicate, so quantile normalization was used to assure values from the replicates were comparable. Data from the replicates were combined using pseudomedian smoothing over a window size of 110 bp. This gives a single expression value per probe for each condition. We then considered all probes overlapping the exonic regions, by at least 50%, of each transcript. The expression level of this transcript was computed as the median of the signal values for all such probes. For inter-sample comparison, we normalized these expression levels by dividing the values by the slide median, i.e. the median of all probes on the array. RNA-seq libraries and sequencing was performed as described [25] for *Harposporium* sp. and *S. marcescens*; for the other pathogens, read lengths were 76 bases rather than 36 bases. Reads were aligned to the WS170 *C. elegans* genome as previously described [25]. To normalize for both transcript length and read number aligned in each sample, the average depth of coverage per million reads (dcpm) was calculated as the total of scores for above threshold bases with representation values of less than 96 divided by the window size and by the number of bases in the transcript unit with a representation value of less than 96, then multiplied by 1,000,000 and divided by the number of high quality mapped reads [25]. All expression data has been submitted to the modENCODE data coordinating center. Analysis algorithms may evolve; readers are referred to the modENCODE site for the latest version of the processed expression data.

### Definition of expressed/detected genes

For the oligo-array results [22], 20,257 genes on the microarrays, had signal strengths twice that of background and ‘unflagged’ data points in at least four out of six microarrays for each pathogen. These were used as the base set for all subsequent analyses. For the cDNA-arrays, probes with expression higher than 40 photostimulated luminescence (PSL) units in the infected or the uninfected sample in at least one of the two experiments [11] were used to define the base set. For RNA-seq, transcripts associated with non-zero dcpm values in at least one of the data sets were used to define the base set. For the tiling array, as there were no zero values for any transcript, all were used to define the base set.

### Definition of up- or down-regulated genes based on fold change

For RNA-seq and tiling arrays, log2 fold changes between gene expression values of infected versus uninfected nematodes were calculated. For log2 fold changes >0.00001 the values >81.25th percentile were defined as up-regulated and for log2 fold changes <−0.00001 the values <18.75th percentile were defined as down-regulated. For oligo-arrays, lists of up- and down-regulated genes were taken from [22] (Table S1A) for *S. marcescens, E. faecalis and P. luminescens* data. The relevant data from the publicly available *D. coniospora* data set that we previously generated (Array Express E-MEXP-479) but had not published is given in Table S17. Differentially regulated genes were defined on the basis of fold change, and corresponded to the uppermost 18.75th percentile of data sets formed using genes with normalized, expression ratios (infected/control) >1.01 or <0.99 in at least ten out of fourteen arrays. For cDNA-arrays, for *S. marcescens* data, for genes with expression >40 PSL in data sets I and II [11], the mean fold change of infected versus uninfected was calculated. If expression was >40 PSL only in data set I or II, the corresponding fold change was taken into account. Fold changes were log2 transformed. For log2 fold changes >0.00001 the values >81.25th percentile were defined as up-regulated and for log2 fold changes <−0.00001 the values <18.75th percentile were defined as down-regulated. For cDNA-arrays, for *D. coniospora* data, lists of up- and down-regulated genes were taken from Table S1A from [12].

### WormBase Converter

Changes in gene annotation and gene structure occur between different versions of WormBase. This hampers a precise comparison of data sets obtained using different WormBase versions for annotation. The WormBase Converter addresses this problem by maintaining a continuously up-dated list of all annotation changes, using the standard WB ID. Any input list of genes, using other gene identifiers (e.g. sequence or transcript names) is first converted to a list of WB ID gene identifiers, using a correspondence table from the appropriate WormBase release. The tool then takes into account all changes in gene annotation that occurred between different versions of WormBase (e.g. genes suppressed, split or merged) and modifies the corresponding WB ID gene identifiers in the list of genes. As many researchers are not familiar with WB ID gene identifiers, but know public gene names, or sequence names, these formats can be chosen for the output list. Furthermore, all changes that occurred during the conversion are displayed in a list to enable tracking of genes that are changed or deleted in the output release. To overcome the fact that many publications that include lists of *C. elegans* genes do not mention the WormBase release used for data annotation, we included the possibility of identifying the most likely WormBase release for a given input list of genes. Lastly, published gene lists often contain a heterogeneous mix of gene identifiers; WormBase Converter can handle such lists and also displays any gene name for which an associated WB ID gene identifier cannot be found. Furthermore, if a new WormBase version is released, the tool automatically retrieves the new data from the server this making it easy to keep lists up to date. WormBase Converter runs under most operating systems (Linux, Windows, Mac OS).

### Ease

We used the freely available software package EASE [32], a user-customized application that performs a statistical analysis to identify significant overrepresentation of functional gene classifications within gene lists. A list of bibliographic annotations was created by adding unpublished gene expression data from our lab and published lists of genes regulated under specific conditions. This list was used for the “bibliographic” functional analysis with EASE of the gene lists regulated upon infection with the different pathogens that we had established from the gene expression data. Lists of gene ontology (GO) annotations were created by downloading GO annotations for all genes from the GO database using an SQL script. We only took into account the most distant annotation and created separate files for the GO annotations “biological process”and “molecular function”. In common with other such tools, EASE suffers from the problem that with changing gene names, functional annotations culled from the literature rapidly become out-dated. To address this problem, we devised the application EASE Manager, based on the algorithms used by WormBase Converter, to maintain an EASE bibliographic database up-to-date, as well as to facilitate the entry of new gene lists from the literature. This application (compatible with the client-server version of the WormBase Converter) and our extensive EASE bibliographic database are freely available at http://wormbasemanager.sourceforge.net/.

### Statistics and figures

Graphpad Prism 5 was used for creating figures and statistical analysis. The Venn diagram generator (http://www.pangloss.com/seidel/Protocols/venn.cgi) and the Venn diagram plotter (http://ncrr.pnl.gov/software/ ) were used to create Venn diagrams and proportional Venn diagrams, respectively.

### Alternative approaches to define up- or down-regulated genes

For both approaches, the expression values are first log10 transformed. The first method that is described in the legend to Figure S3 was coded as a python script. The user defines the number (n) of equal bins into which the data is partitioned, and the cut-off (generally between 0.5 and 10%) applied to these ranked values to define upper and lower y limits to each bin. The script reads three column CSV files (gene name, X, Y) and outputs a graph and two lists of selected genes (up- and down-regulated) as text files. The script is freely available on request. In the second approach, instead of a simple rotation, first the individual data points x_n_ and y_n_, are ranked. Then the average y values for those genes with an x value in the interval between the 20^th^ to 30^th^, and 70^th^ to 80^th^ percentiles is calculated. A line is then drawn through the point represented by the 25^th^ percentile x value and the associated average y value, and the point represented by 75^th^ percentile x value and its associated average y value. The values x and y within a defined bin, orthogonal to this line, and centered at the 25^th^ percentile value, are then projected onto a line through the 25^th^ percentile value. The distribution of these values is calculated and the 99^th^ percentile value defined. A similar procedure is performed around the 75^th^ percentile x value and its associated average y value. A line is then drawn through the two 99^th^ percentile values just defined, and all data points above this line taken to be the differentially regulated genes of interest. At the same time, the geometric distance of each experimental point (i.e. gene) from the line joining the 99^th^ percentiles can be calculated, giving an estimate of the degree to which a given gene represents an outlier in the data. Equivalent calculations can also be performed to identify down-regulated genes.

### Conservation

A set of transcripts (WS170) conserved between nematode species was retrieved from the cisRED database (www.cisred.org) and converted to genes (WS170) using the WormBase Converter. All transcripts with expression in the RNA-seq data set were also converted to genes (WS170). The chi-square test was used to compare the numbers of up-regulated genes after infection with the different pathogens that are conserved to the percentage of conserved genes among the expressed genes.

## Supporting Information

Figure S1 **Changes in gene predictions affect experimental annotation.** Screen shot from WormBase genome browser (http://wormbase.org/db/gb2/gbrowse/c_elegans/), genomic coordinates chromosome III: 7711900 to7717600, showing gene models, obsolete gene models, RNAi clones and microarray probes according to WS222. The gene C03B8.1 has undergone a merge with C03B8.3. The microarray probe cea2.i.18533 was associated with the gene C03B8.1 until WS216 but is associated with gene C03B8.3 from WS217 on.
(PDF)

Figure S2 **Transcripts differentially expressed after *S. marcescens* infection.** Expression of transcripts in uninfected (x-axis) versus *S. marcescens* infected (y-axis) worms. Dot plot showing log10 transformed dcpm values obtained by RNA-seq. (A) Transcripts up- or down-regulated in tiling arrays, RNA-seq and cDNA-arrays are highlighted in red or green, respectively. (B) Transcripts up- or down-regulated in tiling arrays and RNA-seq are highlighted in red or green, respectively. (C) Transcripts up-regulated in tiling arrays and RNA-seq with low expression but high induction are highlighted in red.
(PDF)

Figure S3 **Alternative approach of defining up- and down-regulated transcripts.** (A) Dot plot showing log10 dcpm values for expression of transcripts in uninfected worms (x-axis) versus *D. coniospora* infected worms (y-axis). (B) Dot plot after 45 degree clockwise rotation. The rectangles separate the bins and define upper and lower y limits to each bin. The central x value on the limit y line is marked with a small square. (C) A hyperbole is then fitted to these small squares. Transcripts whose data points lie outside of the hyperbole are defined as up-regulated (red) or down-regulated (blue).
(PDF)

Figure S4 **Overlap of transcripts defined as up-regulated by three methods.** Dot plots showing log10 transformed dcpm values for expression of transcripts in uninfected (x-axis) versus *D. coniospora* infected worms (y-axis). Transcripts identified as up-regulated using three approaches (log2 fold change greater than the 97th percentile of all up-regulated transcripts, and alternative approaches 1 and 2, see Methods) are shown in black; the other transcripts are shown in grey.
(PDF)

Table S1 **Expression of transcripts after infection with *S. marcescens* for 24 hours using tiling arrays.** Public gene, sequence and transcript names come from WS170. The values in the columns Db10 and OP50 are the log2 transformed ratios of the perfect-match to mismatch probe intensities, mapped on to individual transcripts (see Methods) for samples from worms infected for 24 h with *S. marcescens* Db10, or age-matched uninfected control worms. Transcripts identified as up- or down-regulated by infection (see Methods) are indicated.
(XLS)

Table S2 **Expression of transcripts after infection with *S. marcescens* for 24 hours using RNA-seq.** Public gene, sequence and transcript names come from WS170. The values in the columns Db10 and OP50 are the dcpm values calculated for each individual transcript (see Methods) for samples from worms infected for 24 h with *S. marcescens* Db10, or age-matched uninfected control worms. Transcripts identified as up- or down-regulated by infection (see Methods) are indicated. “nan” signifies that there were zero bases in the denominator (see Methods).
(XLS)

Table S3 **Expression of transcripts after infection with *E. faecalis* for 24 hours using tiling arrays.** Public gene, sequence and transcript names come from WS170. The values in the columns EF and OP50 are the log2 transformed ratios of the perfect-match to mismatch probe intensities, mapped on to individual transcripts (see Methods) for samples from worms infected for 24 h with *E. faecalis* strain OG1RF, or age-matched uninfected control worms. Transcripts identified as up- or down-regulated by infection (see Methods) are indicated.
(XLS)

Table S4 **Expression of transcripts after infection with *E. faecalis* for 24 hours using RNA-seq.** Public gene, sequence and transcript names come from WS170. The values in the columns EF and OP50 are the dcpm values calculated for each individual transcript (see Methods) for samples from worms infected for 24 h with *E. faecalis* strain OG1RF, or age-matched uninfected control worms. Transcripts identified as up- or down-regulated by infection (see Methods) are indicated. “nan” signifies that there were zero bases in the denominator (see Methods).
(XLS)

Table S5 **Expression of transcripts after infection with *P. luminescens* for 24 hours using tiling arrays.** Public gene, sequence and transcript names come from WS170. The values in the columns PL and OP50 are the log2 transformed ratios of the perfect-match to mismatch probe intensities, mapped on to individual transcripts (see Methods) for samples from worms infected for 24 h with *P. luminescens* strain Hb, or age-matched uninfected control worms. Transcripts identified as up- or down-regulated by infection (see Methods) are indicated.
(XLS)

Table S6 **Expression of transcripts after infection with *P. luminescens* for 24 hours using RNA-seq.** Public gene, sequence and transcript names come from WS170. The values in the columns PL and OP50 are the dcpm values calculated for each individual transcript (see Methods) for samples from worms infected for 24 h with *P. luminescens* strain Hb, or age-matched uninfected control worms. Transcripts identified as up- or down-regulated by infection (see Methods) are indicated. “nan” signifies that there were zero bases in the denominator (see Methods).
(XLS)

Table S7 **Expression of transcripts after infection with *D. coniospora* for 12 hours using RNA-seq.** Public gene, sequence and transcript names come from WS170. The values in the columns DC and OP50 are the dcpm values calculated for each individual transcript (see Methods) for samples from worms infected for 12 h with *D. coniospora*, or age-matched uninfected control worms. Transcripts identified as up- or down-regulated by infection (see Methods) are indicated. “nan” signifies that there were zero bases in the denominator (see Methods).
(XLS)

Table S8 **Expression of transcripts after infection with *Harposporium* sp. for 24 hours using RNA-seq.** Public gene, sequence and transcript names come from WS170. The values in the columns Harposporium and OP50 are the dcpm values calculated for each individual transcript (see Methods) for samples from worms infected for 24 h with *Harposporium* sp., or age-matched uninfected control worms. Transcripts identified as up- or down-regulated by infection (see Methods) are indicated. “nan” signifies that there were zero bases in the denominator (see Methods).
(XLS)

Table S9 **Lists of differentially regulated genes.** Lists of up- or down-regulated genes (WS210) after infection with different pathogens, established by RNA-seq (A), tiling arrays (B), oligo-arrays (C) and cDNA-arrays (D).
(XLS)

Table S10 **Genes regulated by bacterial infection.** Lists of genes up-regulated by infection with bacterial pathogens (RNA-seq) (A). Genes up-regulated by infection with all three bacterial pathogens (RNA-seq) (B). Lists of genes down-regulated by infection with bacterial pathogens (RNA-seq) (C). Genes down-regulated by infection with all three bacterial pathogens (RNA-seq) (D). Gene lists are based on WS210.
(XLS)

Table S11 **Gene categories regulated by bacterial infection.** Shared gene categories up- (A) or down-regulated (B) upon bacterial infection (RNA-seq).
(XLS)

Table S12 **Genes regulated by bacterial and/or fungal infection.** Lists of genes up- (A) or down-regulated (B) by infection with fungal and bacterial pathogens (RNA-seq). Genes up-regulated by infection with two fungal and three bacterial pathogens (RNA-seq) (C), with *Harposporium* sp. and *D. coniospora* (RNA-seq) (D), with *Harposporium* sp. and three bacterial pathogens (RNA-seq) (E). Genes down-regulated by infection with *Harposporium* sp. and *D. coniospora* (RNA-seq) (F), with *Harposporium* sp. and three bacteria (RNA-seq) (G). Gene lists are based on WS210.
(XLS)

Table S13 **Gene categories regulated by bacterial and/or fungal infection.** Shared gene categories up (A) and down-regulated (B) upon fungal and bacterial infection (RNA-seq).
(XLS)

Table S14 **Overlap between platforms of genes up-regulated after infection.** Numbers of *C. elegans* genes detected as up-regulated after *S. marcescens* (A), *P. luminescens* (B), *E. faecalis* (C) and *D. coniospora* (D) infection. The bold values along the diagonal indicate the total number of genes classed as up-regulated using the different experimental techniques. The off-diagonal values indicate the numbers or percentages of genes out of each total that were also found using the two techniques. For each technique, the number of genes included in the analysis (see Methods) is shown in parenthesis (A).
(PDF)

Table S15 **Genes regulated by S. marcescens infection.** Genes up-regulated after *S. marcescens* infection: genes shared between data sets (A). Potential biomarkers for *S. marcescens* infection (B). Gene lists are based on WS210.
(XLS)

Table S16 **Genes regulated by *E. faecalis* or *P. luminescens* infection.** Genes up-regulated after *E. faecalis* (A) or *P. luminescens* (B) infection: genes shared between data sets. Gene lists are based on WS210.
(XLS)

Table S17 **Expression of genes after infection with *D. coniospora* for 12 hours using oligo-arrays.** The table was generated from previously unpublished data, publicly available at Array Express (E-MEXP-479). Median ratio of gene expression in infected versus uninfected nematodes (A). Genes up- or down-regulated after *D. coniospora* infection (B). CDS sequence names are based on WS150.
(XLS)

Table S18 **Genes regulated by *D. coniospora* infection.** Genes (WS210) up-regulated after *D. coniospora* infection (12 h): genes shared between data sets.
(XLS)
