# Supplementary figures and images for: A Comprehensive Analysis of Gene Expression Changes Provoked by Bacterial and Fungal Infection in C. elegans
Source: PLoS One. 2011 May 13;6(5):e19055. doi: 10.1371/journal.pone.0019055 (PMC3094335; doi:10.1371/journal.pone.0019055)

Figure S1

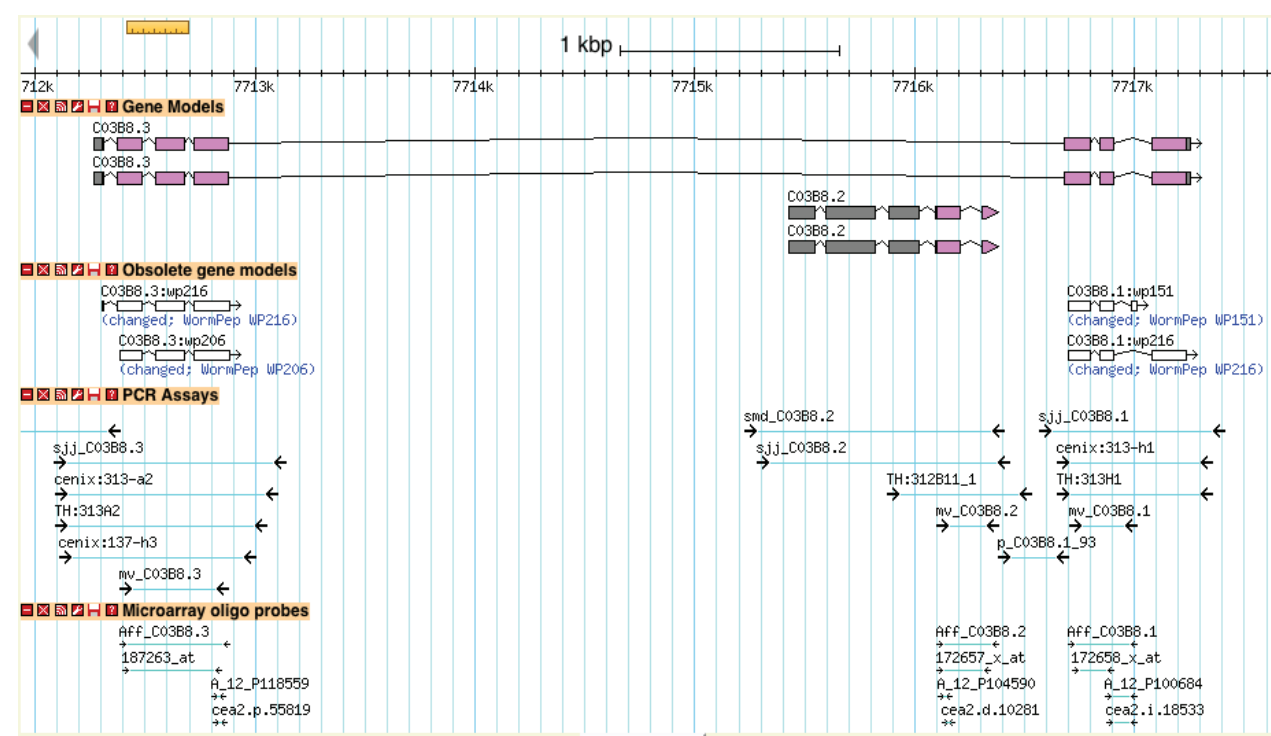

Supplement: Figure S1 — Changes in gene predictions affect experimental annotation. Screen shot from WormBase genome browser (http://wormbase.org/db/gb2/gbrowse/c_elegans/), genomic coordinates chromosome III: 7711900 to7717600, showing gene models, obsolete gene models, RNAi clones and microarray probes according to WS222. The gene C03B8.1 has undergone a merge with C03B8.3. The microarray probe cea2.i.18533 was associated with the gene C03B8.1 until WS216 but is associated with gene C03B8.3 from WS217 on. (PDF) [file pone.0019055.s001.pdf]

**Figure S2**

**A**

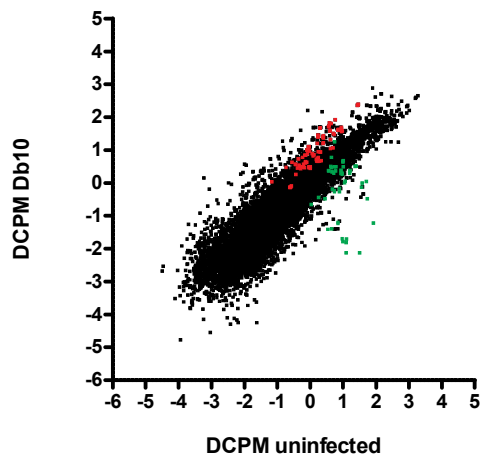

**B**

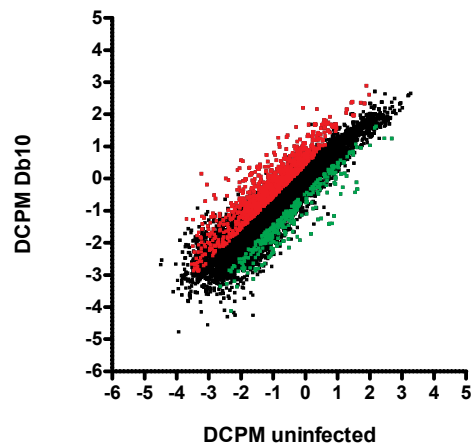

**C**

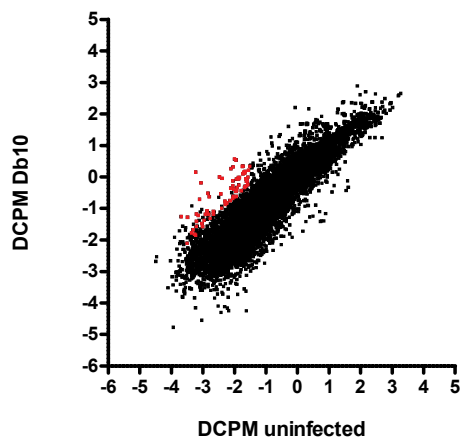

Supplement: Figure S2 — Transcripts differentially expressed after S. marcescens infection. Expression of transcripts in uninfected (x-axis) versus S. marcescens infected (y-axis) worms. Dot plot showing log10 transformed dcpm values obtained by RNA-seq. (A) Transcripts up- or down-regulated in tiling arrays, RNA-seq and cDNA-arrays are highlighted in red or green, respectively. (B) Transcripts up- or down-regulated in tiling arrays and RNA-seq are highlighted in red or green, respectively. (C) Transcripts up-regulated in tiling arrays and RNA-seq with low expression but high induction are highlighted in red. (PDF) [file pone.0019055.s002.pdf]

Figure S3

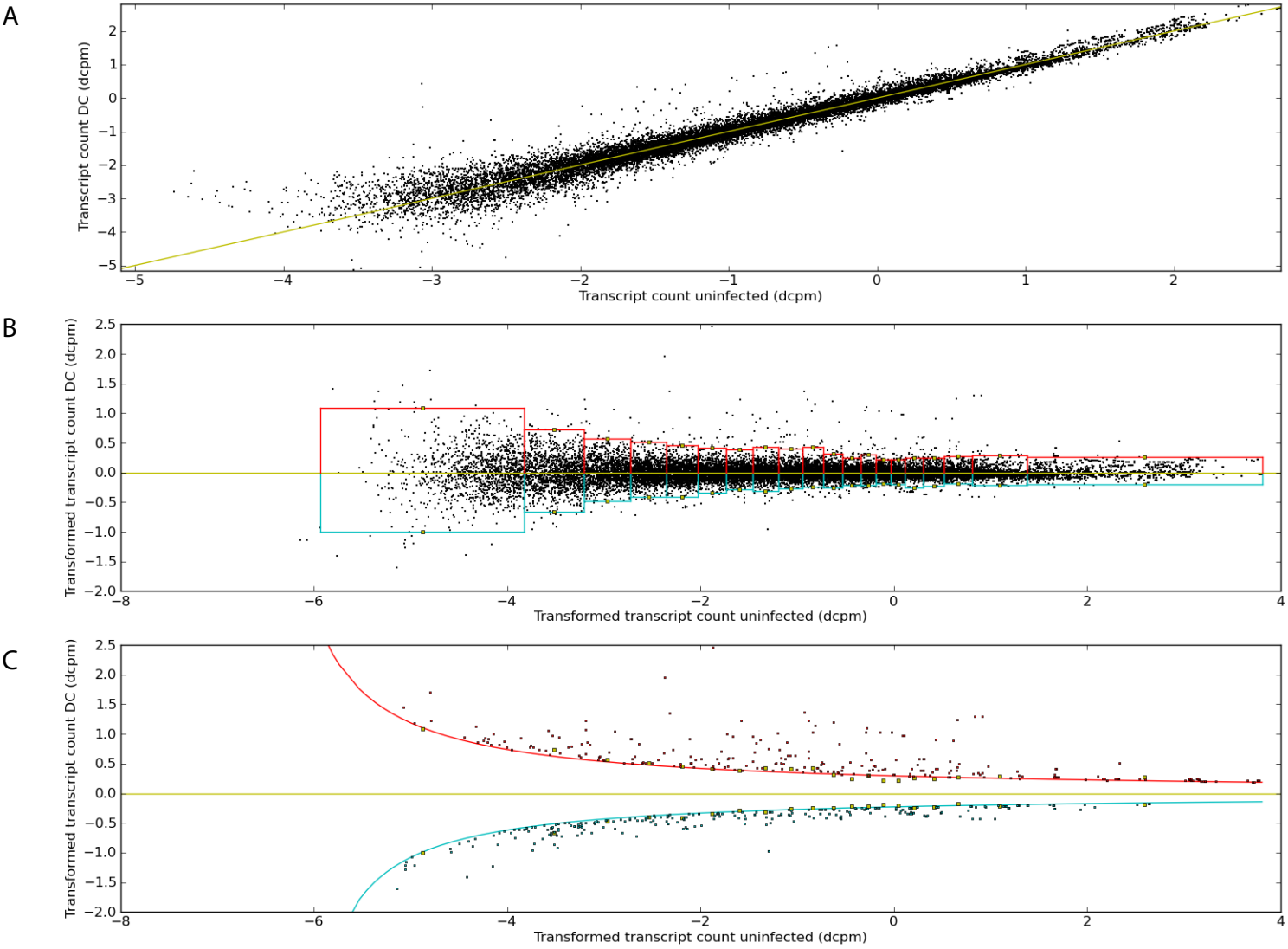

Supplement: Figure S3 — Alternative approach of defining up- and down-regulated transcripts. (A) Dot plot showing log10 dcpm values for expression of transcripts in uninfected worms (x-axis) versus D. coniospora infected worms (y-axis). (B) Dot plot after 45 degree clockwise rotation. The rectangles separate the bins and define upper and lower y limits to each bin. The central x value on the limit y line is marked with a small square. (C) A hyperbole is then fitted to these small squares. Transcripts whose data points lie outside of the hyperbole are defined as up-regulated (red) or down-regulated (blue). (PDF) [file pone.0019055.s003.pdf]

Figure S4

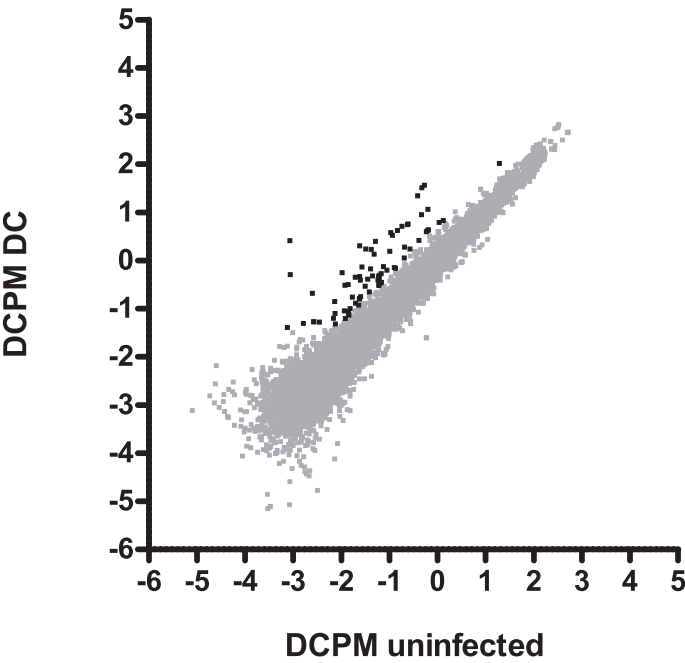

Supplement: Figure S4 — Overlap of transcripts defined as up-regulated by three methods. Dot plots showing log10 transformed dcpm values for expression of transcripts in uninfected (x-axis) versus D. coniospora infected worms (y-axis). Transcripts identified as up-regulated using three approaches (log2 fold change greater than the 97th percentile of all up-regulated transcripts, and alternative approaches 1 and 2, see Methods) are shown in black; the other transcripts are shown in grey. (PDF) [file pone.0019055.s004.pdf]
