# Supplementary material for: A Comprehensive Analysis of Gene Expression Changes Provoked by Bacterial and Fungal Infection in C. elegans
Source: PLoS One. 2011 May 13;6(5):e19055. doi: 10.1371/journal.pone.0019055 (PMC3094335; doi:10.1371/journal.pone.0019055)
Supplement: Table S14 — Overlap between platforms of genes up-regulated after infection. Numbers of C. elegans genes detected as up-regulated after S. marcescens (A), P. luminescens (B), E. faecalis (C) and D. coniospora (D) infection. The bold values along the diagonal indicate the total number of genes classed as up-regulated using the different experimental techniques. The off-diagonal values indicate the numbers or percentages of genes out of each total that were also found using the two techniques. For each technique, the number of genes included in the analysis (see Methods) is shown in parenthesis (A). (PDF) [file pone.0019055.s018.pdf]

Table S14

A

Technique (number of genes detected)	RNA-seq (17 500)	Tiling array (20 081)	Oligo array (20 257)	cDNA array (1527)
**RNA-seq**	**2276**	1008	63	56
**Tiling array**	44%	**2661**	80	55
**Oligo array**	11%	14%	**588**	6
**cDNA array**	34%	33%	4%	**167**

B

Technique	RNA-seq	Tiling array	Oligo array
**RNA-seq**	**3037**	1371	160
**Tiling array**	45%	**3813**	190
**Oligo array**	25%	29%	**646**

C

Technique	RNA-seq	Tiling array	Oligo array
**RNA-seq**	**3123**	1030	141
**Tiling array**	33%	**4088**	174
**Oligo array**	22%	28%	**633**

D

Technique	RNA-seq	Oligo array	cDNA array
**RNA-seq**	**1533**	33	16
**Oligo array**	8%	**402**	18
**cDNA array**	6%	7%	**265**
